# Automated Segmentation Methods of Drusen to Diagnose Age-Related Macular Degeneration Screening in Retinal Images

**DOI:** 10.1155/2018/6084798

**Published:** 2018-03-12

**Authors:** Young Jae Kim, Kwang Gi Kim

**Affiliations:** Department of Biomedical Engineering, Gachon University College of Medicine, Incheon, Republic of Korea

## Abstract

Existing drusen measurement is difficult to use in clinic because it requires a lot of time and effort for visual inspection. In order to resolve this problem, we propose an automatic drusen detection method to help clinical diagnosis of age-related macular degeneration. First, we changed the fundus image to a green channel and extracted the ROI of the macular area based on the optic disk. Next, we detected the candidate group using the difference image of the median filter within the ROI. We also segmented vessels and removed them from the image. Finally, we detected the drusen through Renyi's entropy threshold algorithm. We performed comparisons and statistical analysis between the manual detection results and automatic detection results for 30 cases in order to verify validity. As a result, the average sensitivity was 93.37% (80.95%~100%) and the average DSC was 0.73 (0.3~0.98). In addition, the value of the ICC was 0.984 (CI: 0.967~0.993, *p* < 0.01), showing the high reliability of the proposed automatic method. We expect that the automatic drusen detection helps clinicians to improve the diagnostic performance in the detection of drusen on fundus image.

## 1. Introduction

Age-related macular degeneration (AMD) is a significant cause of visual impairment in the United States. AMD affects more than 1.75 million individuals in the United States, and it is estimated that this number will increase to 3 million by 2020 [1]. The incidence of AMD will continue to rise with the growing elderly population, resulting in visual disability, a decrease in quality of life, and an increase in the risk of falls, fractures, depression, and mortality [2, 3].

Macular degeneration is induced by drusen stored inside or outside of the retina pigmentary epithelium [4]. Drusen falls into two types: hard drusen or soft drusen. Hard drusen which is able to progress to soft drusen can be found in all age groups and is largely irrelevant to macular degeneration. Soft drusen which develops choroidal neovascularization causing visual loss can be found among the elderly and is relevant to macular degeneration [5]. As the macular degeneration still lacks effective treatment, the prevention is important: prevention of the progression of hard drusen into soft drusen by continuous measurement. Therefore, quantitative measurement of drusen is very important to prevent macular degeneration. However, manual drusen measurement based on existing visual inspection requires a lot of time and effort. In addition, the influence of subjective decision makes manual measurement less reliable.

In this study, we aim to solve the problems of manual measurement by developing an automatic drusen segmentation method based on computer-aided diagnosis (CAD). CAD is various image-processing techniques used in performing difficult measurements [6, 7]. An ophthalmologist can save time by drusen automatic measurements. Additionally, results have a high reproducibility and reliability through objective and quantitative measurements. In this paper, we developed an algorithm that accurately segments drusen in fundus image using the median filter and Renyi's threshold algorithm. Moreover, we verified the algorithm by comparing results from the manual segmentation method.

## 2. Materials and Methods

For our study, we collected 48 fundus images with ARMD from the Seoul National University Bundang Hospital. Of these 30 fundus images, 15 were captured from the left while 15 were captured from the right. Test image data have widths of 1536 pixels and heights of 1024 pixels in a 32-bit RGB color. Programs used in the experiment were Microsoft Visual Studio (Ver. 2010, Microsoft, Redmond, WA, USA), ITK (Insight Segmentation and Registration Toolkit, Kitware Inc., NY, USA), and VTK (Visualization Toolkit, Kitware Inc., NY, USA).

## 3. Methods

**Figure 1:**
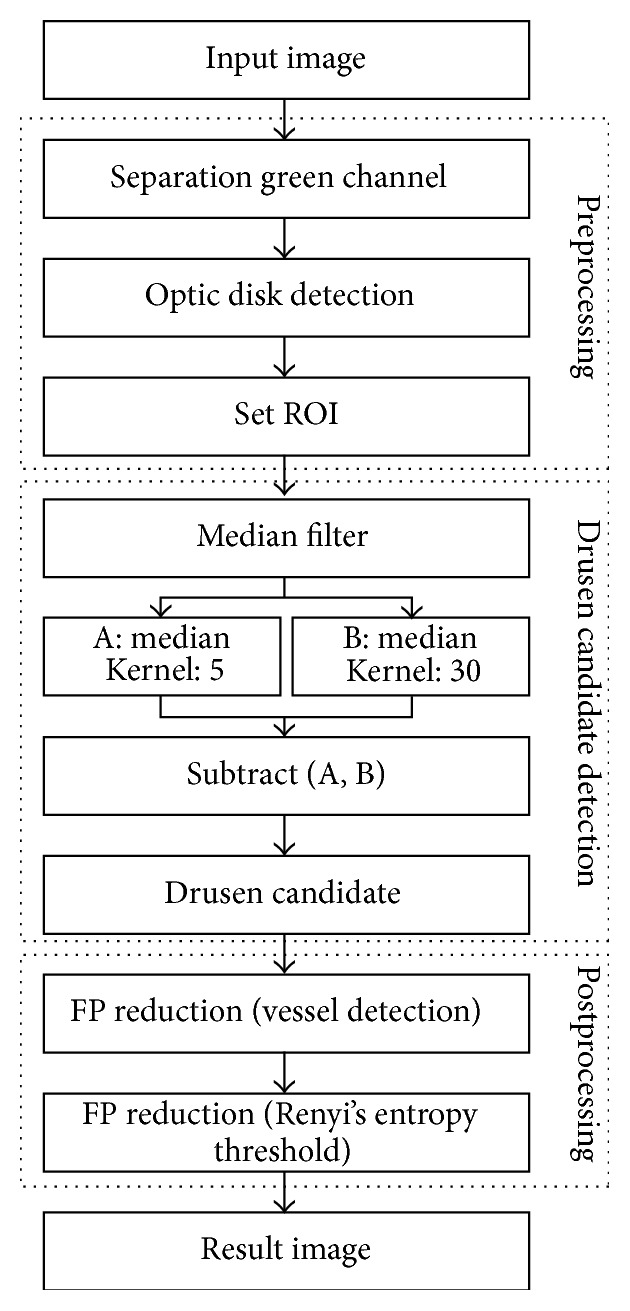
Flow chart of the proposed algorithm.

The automatic measurement method of drusen proposed in this paper mainly consists of preprocessing, drusen candidate detection, and postprocessing tasks.  Figure 1 shows the complete flowchart of the algorithm that this paper proposes.

### 3.1. Preprocessing

#### 3.1.1. Color Split

In order to detect drusen, we analyzed the color space, which shows drusen the most among all other color spaces of the images: RGB (red-green-blue), HIS (hue-saturation-intensity), CMYK (cyan-magenta-yellow-black), G/R and R/B (green/red, red/blue bands), and so forth. Our empirical observations provide the selection of the green channel inside, as the channel with the maximum contrast in the RGB space. The green band contains useful information, as the channel holds the maximum contrast in the RGB space. The red band contains useful information for reflectance in the image and therefore is strongly affected by the nonuniform illumination, while the blue band relatively provides less information, which is not useful for drusen detection. The green band is much more informative for drusen and less affected from the overall variation of illumination [8]. Thus, this paper used the green filter image in RGB space.

#### 3.1.2. Region of Interest (ROI)

ROI were placed inside where drusen mostly appears and inside where drusen affects visual acuity the most. This paper used the automatic drusen detection method while ROI converges on circle whose radius connects from fovea to optic disk that the ophthalmologists considers important.

**Figure 2:**
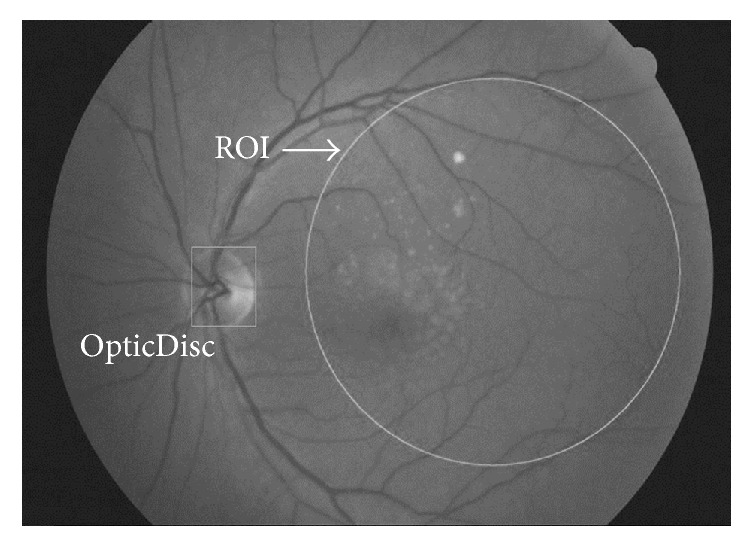
Examples of the optic disc and the ROI detection results. Drusen detection is performed only within the ROI.

We detected optic disk in order to select the ROI. The optic disk is beginning part of optic nerve where the brightest points are on the fundus image. Thus, its detection is possible only by threshold. However, the brightest location on 8-bit images having intensity of 256 is not enough for detection; we labeled the location after threshold. The optic disk can also have similar pixels intensity, so in this paper we propose an accurate detection through threshold and labeling. We have labeled a high intensity of more than 200 because optic disk possesses the brightest intensity in the images, and we have assumed the largest label as an optic disc. The intensity values of 200 were empirically determined through experiments with various values. We calculated median pixel point using optic disk label and traced the location of optic disk which part they may be, left or right from the center of the image. Finding out the location, we measured the distance between the median pixel point of the optic disk and the very end of the image against the optic disk. We determined the midpoint of the distance as the median pixel point of ROI and set up the area of ROI in sphere.  Figure 2 shows examples of the optic disc and the ROI detection results.

### 3.2. Drusen Candidate Detection

**Figure 3:**
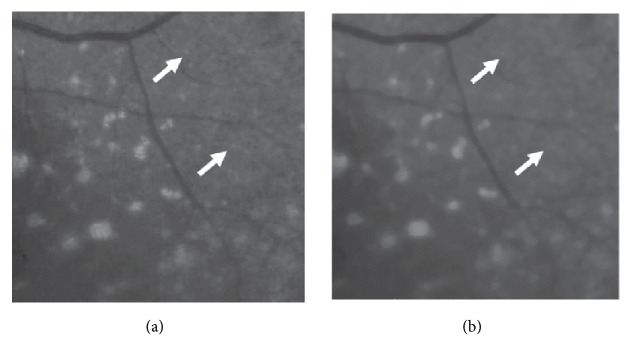
Examples of results before and after applying a small mask-sized median filter for noise reduction. In (a), a salt-type noise is shown where the arrow points, but in (b) the noise is removed and it is invisible. (a) The image before applying median filter; (b) the image after applying median filter.

To detect drusen candidate, median filter is applied. The median filter preserves radical changes as a nonlinear filtering method which is effective against impulse noise [9]. It arranges mask area measurements according to size and selects the median. Hard drusen is characterized by the shape of small spots. We applied a median filter using a larger size mask than the size of the dressing. In applying images, all dressing were disappeared. We experimented with masks of various sizes and decided that 30 × 30 masks were appropriate. We can find drusen location through differences between achieving image and original one. However, not to affect drusen detection due to noise in the original image, we made, subtract image using noise removed image applied the median filter being small sized mask which affects only noise except dressing. We experimented with masks of various sizes and determined that it is appropriate to remove the noisy without affecting drusen in a 5 × 5 mask.  Figure 3 shows the results before and after applying a small mask size median filter for noise reduction. The result shows that the drusen has not been removed, but the small impulse noise has been removed.

**Figure 4:**
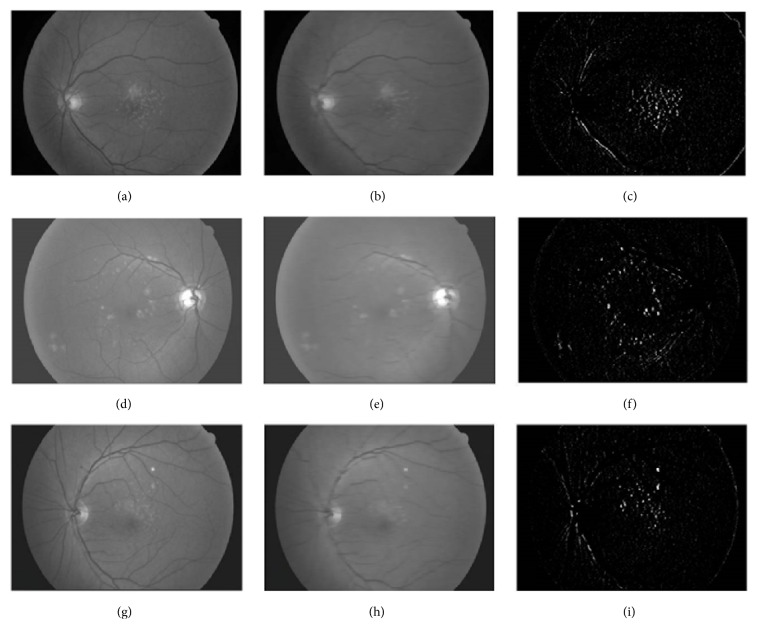
Examples of different median filter results. (a), (d), (g) Results of median filter, the size of the mask is 5. (b), (e), (h) Results of median filter, the size of the mask is 30. (c) Results of subtract image between (a) and (b). (f) Result of subtract image between (d) and (e). (i) Result of subtract image between (g) and (h).

Figure 4 presents pair of images being measured by different median filter and the subtract image between the two. The subtract images have their contrast concentrated in the low area. However, we make histogram spread throughout gray scale using histogram stretching so that contrast can be higher. In the subtract image, result is determined by blood vessel, optic disk, background, and drusen. When getting rid of these nondrusen areas one by one, final drusen detection is performed in this paper.

### 3.3. Postprocessing (FP Reduction)

In the subtract images using median filter, pixel intensity changes according to the brightness difference between two images. Drusen that is much brighter than other areas has high pixel intensity. Background pixels in both two images has little differences being 0 or lower intensity. Among the detected candidates, some having high pixel intensity can be displayed around drusen, optic disk, and blood vessel which has high intensity than background. In this paper, a two-step approach is proposed beginning with detection of drusen, optic disk, and blood vessel candidates. Then, it proposed the detection of nondrusen, which appears around the optic disk and vessels to detect drusen.

#### 3.3.1. Vessel Detection

In the subtract images, nondrusen candidates can be found nearby optic disk and vessel which has high intensity compared to background. A first approach to this problem was detecting optic disk and vessels. We also eliminated irregularities in the result image. Optic disk could be found when ROI was placed. Vessel segmentation was proposed using optic disk coordinates and region growing method for its high pixel intensity [10]. Seed point was implanted inside optic disc area: all vessels are sprawling out from optic disk, so the same pixel intensity with blood vessel was determined as seed point and we performed region growing.

#### 3.3.2. Threshold

Among candidates in subtracted images, low pixel intensity background was removed using automatic threshold technique. Automatic threshold technique comes in many different algorithm threshold: Otsu threshold [11, 12], Renyi's entropy threshold [13, 14], maximum entropy threshold, minimum cross-entropy threshold, Yen threshold, and so forth.

**Figure 5:**
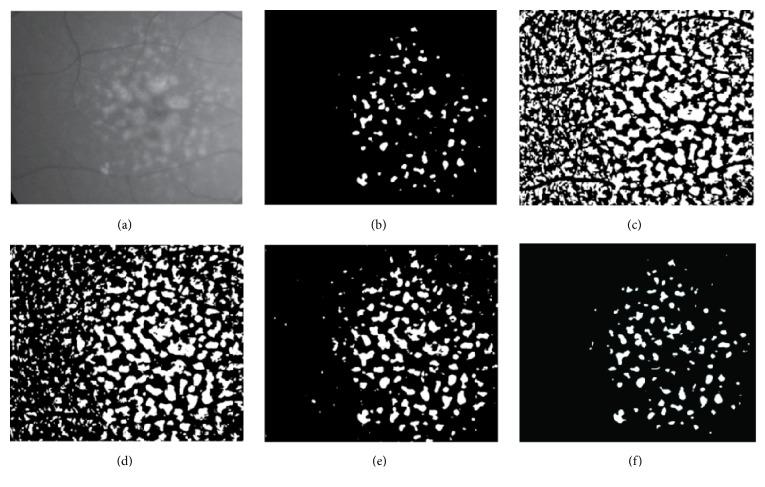
Examples of comparison results of the various automatic threshold methods. (a) Original image, (b) maximum entropy threshold, (c) minimum cross-entropy threshold, (d) Otsu threshold, (e) Yen threshold, and (f) Renyi's entropy threshold.

In this paper we present Renyi's entropy method in threshold. We applied various automatic threshold methods to the data, and Renyi's entropy method showed better results.  Figure 5 shows the comparison of various automatic threshold methods.

Renyi's entropy threshold method [13] uses two probability distributions, which are object of interest in an image and background based on their gray-level distribution. Let *P*_0_, *P*_1_, *P*_2_,…, *P*_255_, be the gray value of the probability distribution. The object of interest *A*_1_ and background *A*_2_ are calculated as

(1) PA1=∑i=0tPi,PA2=∑i=t+1255Pi,PA1+PA2=1.

Renyi's entropy in image *α* is defined as follows:

(2) $$\begin{array}{c} H_{T}^{\alpha }=\frac{1}{1-\alpha }\ln\overset{255}{\underset{k=0}{\sum }}{\left(\;P_{k}\;\right)}^{\alpha }. \end{array}$$

*α* in formula (2) means real number, *α*  (≠1), Renyi's entropy *H*_*T*_^*α*^ converges on Shannon entropy *H*_*T*_ according to lim⁡*α* → 1  *H*_*T*_^*α*^ = *H*_*T*_. Renyi's entropy associated with object of interest and background distributions can be given by

(3) HA1αt=11−αln⁡∑i=0tPiPA1α,HA2αt=11−αln⁡∑i=t+1255PiPA2α.

*t*(*α*) represents gray value which is maximum of *H*_*A*_1__^*α*^(*t*) + *H*_*A*_2__^*α*^(*t*) in formula (4). And *t*_1_, *t*_2_, *t*_3_ are determined according to *α* as 3 types of results in formula (5):

(4) tα=arg maxt∈G⁡HA1αt+HA2αt,

(5) tα=t1if  0<α<1t2if  α⟶1t3if  1<α<∞.

When *α* is 1 which is *t*_2_, the result equals the same result found by maximum entropy sum method. When *α* > 1 which is *t*_3_ the result equals the result found by entropic correlation method. The optimal threshold value *t*_*c*_ is calculated by using *t*_1_, *t*_2_, *t*_3_ as the following formula:

(6) tc=t1Pt1+14ωβ1+14t2ωβ2+t31−Pt3+14ωβ3.

*P*(*t*) = ∑_*i*=1_^*t*^*P*_*i*_ and *ω* = *P*(*t*_[3]_) − *P*(*t*_[1]_). (*β*_1_, *β*_2_, *β*_3_) is defined as follows:

(7) β1,β2,β3=1,2,1if  t1−t2≤5,  t2−t3≤51,2,1if  t1−t2>5,  t2−t3>50,1,3if  t1−t2≤5,  t2−t3>53,1,0if  t1−t2>5,  t2−t3≤5.

**Figure 6:**
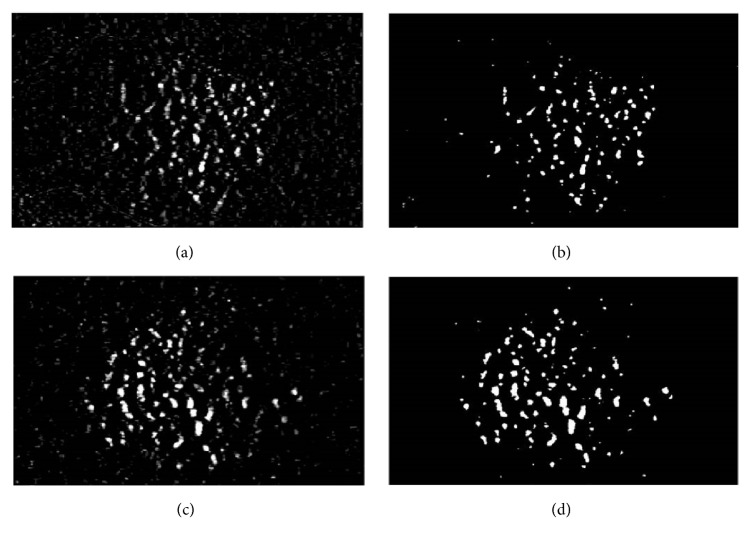
Examples of Renyi's entropy threshold algorithm results. (a), (c) An image of drusen candidate. (b), (d) An image of Renyi's entropy threshold algorithm.

Figure 6 presents the result of the Renyi's entropy thresholding applied to an image containing drusen candidate. We can detect only drusen without presence of noise excluding few areas nearby vessels and surrounding of the image. However, surrounding areas of the image and vessels nearby optic disk are located out of the ROI not being influential on drusen detection.

## 4. Results

**Figure 7:**
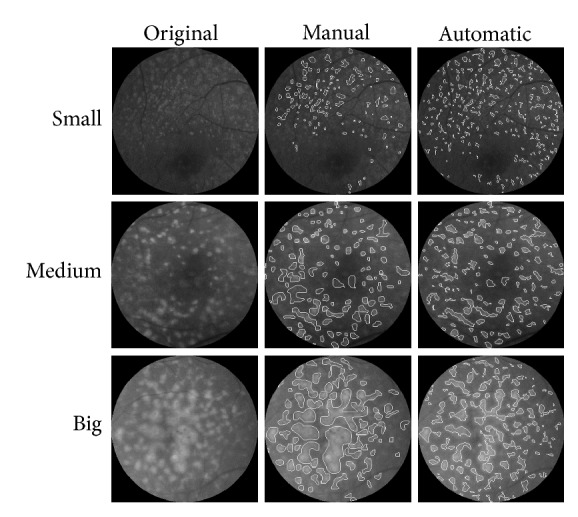
Examples of drusen detection results by size (small, medium, and big size drusen). Comparison between the manual detection method and proposed automatic detection method.

We proposed an automatic drusen detection method using the median filter and Renyi's threshold algorithm. The result is shown in Figure 7.

We performed statistical analysis between the manual segmentation results and automatic segmentation results for 30 cases in order to validation of the proposed method. To obtain the results of manual segmentation, the ophthalmologist segmented drusen using in-house developed software. The statistical analysis used conditional probability (sensitivity, specificity, accuracy, and Dice's Similarity Coefficient (DSC)), correlation analysis, and reliability analysis.

We automatically detected the position of the optic disc and the ROI for the macula in 30 data sets. The position of the detected the optic disc and the ROI was evaluated by the ophthalmologist. As a result, the position of the optic disc and the ROI was detected with 100% accuracy.

In this paper, True Positive (TP), False Positive (FP), True Negative (TN), and False Negative (FN) were calculated by comparing the results from the automatic and manual methods. TP means that the drusen was correctly detected, and FP means that it detects wrong place other than the drusen. TN means that it has not detected a nondrusen, and FN means that the drusen was not detected. These two different calculating methods “pixel by pixel” and “region by region” were used to get the results. “Pixel by pixel” is to compare every pixel in the predefined ROI; “region by region” is to compare depending on the region with computed drusen area.

TP, FP, TN, and FN were calculated by the two methods and the conditional probability was calculated as shown in Table 1. The method of “pixel by pixel” averagely showed 81.93% (67.31%~94.58%) sensitivity, 96.83% (87.09%~99.24%) specificity, 94.82% (86.15%~98.45%) accuracy, and 0.79 (0.68~0.94) DSC. Also, the method of “region by region” averagely showed 93.37% (80.95%~100%) sensitivity and 0.73 (0.3~0.98) DSC. Specificity and accuracy were excluded because it was impossible to compute TN from the method of “region by region.”

**Figure 8:**
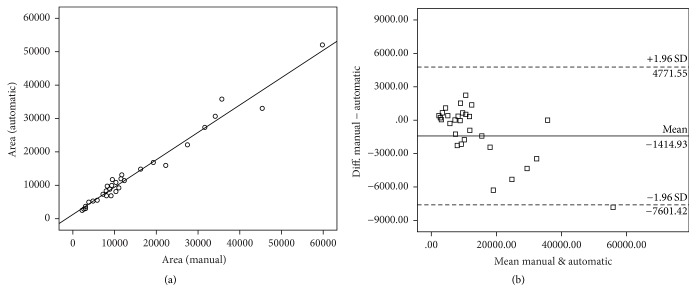
Graph for the correlation between manual and automatic area measurement results. (a) Scatter plots; (b) Bland-Altman plots.

In this paper, correlation analysis and reliability analysis were performed on areas of drusen measured by automatic and manual methods. As a result, the area of drusen measured by automatic method shows the significant correlation (*r* = 0.986, *p* < 0.01) with the area of drusen measured by the manual method in the correlation analysis using Pearson's correlation as shown in Figure 8(a). Additionally, Bland-Altman plots show quite good comparability as shown in Figure 8(b), as most values are within ±1.96 standard deviations from the average position of the respective area differences. In the reliability analysis using Intraclass Correlation Coefficient (ICC) which was used to evaluate the agreement between the two evaluators, ICC was 0.984 (CI: 0.967~0.993, *p* < 0.01) between the areas of drusen measured by manual and automatic methods, and there was no difference in the measured area.

## 5. Discussion

Existing drusen measurement is difficult to use in clinic because it requires a lot of time and effort for visual inspection. In order to resolve this problem, we proposed an automatic method for drusen detection using computer image-processing techniques. The sensitivity of the automatic detection result compared to that of manual detection result is 93.37% and DSC is 0.73. This result shows higher sensitivity than the results of other studies that attempted an automatic drusen detection. For example, Brandon and Hoover attempted to design a statistics method of multilevel classification approach and obtained an average accuracy of 87% with 119 images [15]. Moreover, Köse et al. tried automatic segmentation of drusen by using inverse region growing method and obtained an average accuracy of 92.07% with 30 images [16]. Brandon and Hoover's study showed a relatively low detection rate of 87% detection rate. In addition, since the entire fundus image is used without ROI, it takes a long time for the algorithm and the probability of occurrence of FP increases. On the other hand, we can reduce the processing time of the algorithm and detect more precisely, because we limit the algorithm target to the macula area through the ROI. Köse et al.'s study showed high accuracy. However, it is based on a region growing algorithm. If there is a gradient in the fundus image, it can affect the region growing algorithm. However, because we use the difference image with the gradient removed, there is an advantage that the threshold can be detected accurately without any problem caused by the gradient. The studies compared above were evaluated using different data. Therefore, it was difficult to objectively compare with our studies. In future studies, we will use open-data to evaluate the performance of the proposed method and to perform additional validation by comparing it with other studies using the same data.

Both “pixel by pixel” and “region by region” methods showed high sensitivity (81.93%, 94.82%), whereas DSC (0.79, 0.73) showed relatively low results. It was analyzed that DSC was low because FP was detected much. In addition, soft drusen with a wide spread shape had limitations in detection due to ambiguous boundary. The leading cause of FP was the background noise. We can reduce the FP by adjusting the kernel of the filter, but there is a risk of affecting drusen. Therefore, it is necessary to study algorithms that can remove noise without affecting drusen. We expect it to be effective in reducing FP by using techniques using the features of drusen such as machine learning and deep learning. In further studies, we believe the performance can be improved by developing additional algorithms to solve the problem of FP removal and soft drusen detection. In conclusion, we expect that the automatic drusen detection helps clinicians to improve the diagnostic performance in the detection of drusen on fundus image.

## Figures and Tables

**Table 1:** Statistical analysis results between the manual segmentation and automatic segmentation.

	Sensitivity (%)	Specificity (%)	Accuracy	DSC
Pixel by pixel	81.93	96.83	94.82	0.79
Region by region	93.37	-	-	0.73

Correlation	0.986^*∗*^			
ICC	0.987^*∗*^			

^*∗*^ *p* < 0.01.
